# Delivering non-communicable disease interventions to women and children in conflict settings: a systematic review

**DOI:** 10.1136/bmjgh-2019-002047

**Published:** 2020-04-27

**Authors:** Shailja Shah, Mariella Munyuzangabo, Michelle F Gaffey, Mahdis Kamali, Reena P Jain, Daina Als, Sarah Meteke, Amruta Radhakrishnan, Fahad J Siddiqui, Anushka Ataullahjan, Zulfiqar A Bhutta

**Affiliations:** 1 Centre for Global Child Health, The Hospital for Sick Children, Toronto, Ontario, Canada; 2 Health Services and Systems Research, Duke-NUS Graduate Medical School, Singapore; 3 Center of Excellence in Women and Children Health, Aga Khan University, Karachi, Pakistan

**Keywords:** public health, cardiovascular disease, diabetes, systematic review, maternal health

## Abstract

**Background:**

Non-communicable diseases (NCDs) are the leading cause of death worldwide. In the context of conflict settings, population displacement, disrupted treatment, infrastructure damage and other factors impose serious NCD intervention delivery challenges, but relatively little attention has been paid to addressing these challenges. Here we synthesise the available indexed and grey literature reporting on the delivery of NCD interventions to conflict-affected women and children in low- and middle-income countries (LMICs).

**Methods:**

A systematic search in MEDLINE, Embase, CINAHL and PsycINFO databases for indexed articles published between 1 January 1990 and 31 March 2018 was conducted, and publications reporting on NCD intervention delivery to conflict-affected women or children in LMICs were included. A grey literature search of 10 major humanitarian organisation websites for publications dated between 1 January 2013 and 30 November 2018 was also conducted. We extracted and synthesised information on intervention delivery characteristics and delivery barriers and facilitators.

**Results:**

Of 27 included publications, most reported on observational research studies, half reported on studies in the Middle East and North Africa region and 80% reported on interventions targeted to refugees. Screening and medication for cardiovascular disease and diabetes were the most commonly reported interventions, with most publications reporting facility-based delivery and very few reporting outreach or community approaches. Doctors were the most frequently reported delivery personnel. No publications reported on intervention coverage or on the effectiveness of interventions among women or children. Limited population access and logistical constraints were key delivery barriers reported, while innovative technology use, training of workforce and multidisciplinary care were reported to have facilitated NCD intervention delivery.

**Conclusion:**

Large and persistent gaps in information and evidence make it difficult to recommend effective strategies for improving the reach of quality NCD care among conflict-affected women and children. More rigorous research and reporting on effective strategies for delivering NCD care in conflict contexts is urgently needed.

**PROSPERO registration number:**

CRD42019125221

**Key questions**

**What is already known?**

- Conflict imposes long-term and intergenerational effects on the health of children that place them at higher risk of future non-communicable diseases (NCDs).
- Little research and programmatic attention have been given to providing NCD-related care to women and children affected by conflict.
- Disrupted and delayed NCD treatment due to conflict may lead to poorer outcomes for patients and increase costs of managing complications for humanitarian agencies.

**What are the new findings?**

- Limited information on the delivery of NCD interventions in conflict contexts is available in the literature, with almost no available data on intervention coverage or effectiveness among women or children.
- Humanitarian actors face logistical and other challenges in accessing conflict-affected populations to screen for NCDs and deliver interventions, especially in the context of population displacement.

**What do the new findings imply?**

- More rigorous research and reporting on effective strategies for delivering NCD care in conflict contexts is urgently needed, given the increasing burden of NCDs globally.
- Greater focus on strengthening cohort monitoring systems to enhance regular access to NCD patients and promote sustainable care is recommended.

## Introduction

Non-communicable diseases (NCDs) are the leading cause of death worldwide, placing a huge burden on individuals, their families and health systems. The NCDs with the greatest global mortality are cardiovascular diseases, chronic respiratory diseases, cancer and diabetes.^1^ The importance of addressing these and other NCDs is acknowledged in the Sustainable Development Goals, which call for measures to reduce mortality from NCDs by 2030.^1^ However, relatively little research and programmatic attention have been given to addressing NCDs in humanitarian situations.

An estimated 70.8 million people globally have been forcibly displaced.^2^ The long-term and intergenerational health effects of conflict on children’s health are evident, and include congenital abnormalities, malnutrition, and risk of chronic diseases such as diabetes, hypertension and cardiovascular disease in adult offspring.^3 4^ Vulnerable conflict-affected populations with the highest NCD burden include refugees from the Middle East and North Africa region fleeing the Syrian crisis to neighbouring countries including Lebanon, Jordan, Iraq and Turkey.^5^ Conflict situations pose considerable challenges for NCD care and treatment. Disrupted and delayed treatment may lead to poor outcomes for patients and impose high costs of managing complications on humanitarian agencies.^3 4^ A more comprehensive approach to NCD management in emergencies is crucial but has been largely neglected in humanitarian response.^4^ The WHO Package of Essential NCDs Interventions (PEN) aims to prevent life-threatening exacerbations of disease and support maintenance of therapy in low resource settings of low- and middle-income countries;^6^ this package may be a good starting point for integrating NCD care into primary healthcare in conflict-affected and other humanitarian settings,^7^ but it is still derived from the adaptation of high-income setting evidence to non-emergency low-income settings, with potentially limited applicability to the diversity of the NCD epidemiology, NCD knowledge and health-seeking behaviours, and health system characteristics in humanitarian settings.^8^

A previous systematic review of NCD interventions in humanitarian settings found the existing evidence base to be extremely limited in both quantity and quality,^9^ and a recent review on the burden of NCDs and access to NCD services among Syrian refugees in neighbouring host countries highlighted the need for innovative service delivery models.^10^ Both reviews underscore the need for better understanding of how NCD interventions have been and are being delivered in a range of conflict settings. The aim of the present review is to synthesise the available indexed and grey literature reporting on how NCD interventions are being delivered to these vulnerable populations of women and children, with a specific focus on intervention delivery approaches, barriers and facilitators.

## Methods

This systematic literature review adheres to the Preferred Reporting Items for Systematic Reviews and Meta-Analysis statement^11^ and its protocol is filed with PROSPERO (www.crd.york.ac.uk/prospero/), the international prospective register of systematic reviews.

### Search strategy

We systematically searched MEDLINE, Embase, CINAHL and PsycINFO online databases for indexed journal articles published between 1 January 1990 and 31 March 2018 using search terms that related to (i) women, children or adolescents; (ii) conflict or war; and (iii) non-communicable diseases, and were informed by search strategies employed in previous relevant reviews.^9 12^ The full search syntax for MEDLINE is presented in online supplementary appendix 1. We also sought potentially relevant studies from the reference lists of recently published systematic reviews. In addition to indexed literature, we also searched grey literature published between 1 January 2013 and 30 November 2018 on the websites of 10 major humanitarian organisations who are actively involved in responding to or researching conflict situations: Emergency Nutrition Network, International Committee of the Red Cross, International Rescue Committee, Médecins Sans Frontières (MSF), Save the Children, United Nations Population Fund (UNFPA), United Nations High Commissioner for Refugees (UNHCR), United Nations Children’s Fund (UNICEF), Women’s Refugee Commission and World Vision. We used broad terms for conflict and health interventions tailored to the search functionality of each website. Indexed publications from as early as 1990 were potentially eligible, ensuring that we captured as much literature as possible that describes the delivery of interventions that are contemporarily relevant; however, given the large volume of grey literature available, we elected to include only grey literature published in the previous 5 years, from 2013 or later, in order to be able to feasibly assess this literature.

10.1136/bmjgh-2019-002047.supp1 Supplementary data

### Selection criteria

Eligible publications were limited to those reporting on populations affected by conflict in low- or middle-income countries (LMICs), as classified by the World Bank.^13^ They must have described a non-communicable disease intervention that targeted or included children or adolescents of any age, or women of reproductive age (15 to 49 years) and that was delivered during or within 5 years of cessation of a conflict. Eligible NCD interventions did not include those focussing primarily on mental health, as these interventions are considered in a separate systematic review by the same authors. If ongoing or recent cessation of conflict was not mentioned explicitly in the publication, we referred to online encyclopaedic sources as well as the United Nations Office for the Coordination of Humanitarian Affairs (UN OCHA) website for information on whether and when conflict occurred in the focal country of the publication. In order to identify the most informative resources from the large volume of grey literature available, the same eligibility criteria for indexed literature were applied, with the additional requirement of explicit reporting in the grey literature on the delivery site and personnel for each intervention.

Non-English publications; case reports of single patients; publications reporting on military personnel, refugee populations bound for a high-income country, surgical techniques, economic or mathematical modelling; and editorials and opinion pieces were excluded from our review. Other exclusion criteria included systematic reviews, guidelines, and publications where no specific health intervention was described (eg, prevalence studies).

### Data extraction

From all retrieved indexed records, we eliminated duplicate records using EndNote X7 software^14^ and then imported unique records into Covidence software,^15^ which two reviewers used to independently screen each title and/or abstract for relevance. Discrepancies between reviewers’ decisions were resolved via discussion, or by a third reviewer if necessary. A single reviewer then assessed the full text of each potentially relevant publication to determine eligibility for the review. We applied a similar approach to the grey literature, with two reviewers screening the title of each retrieved publication for relevance, and a single reviewer then assessing the full text of each potentially relevant publication for eligibility.

We sought to extract all relevant qualitative and quantitative information from eligiblelivery, delivery through outreach, and comme publications using a structured, pilot-tested data abstraction tool in REDCap (Research Electronic Data Capture) software.^16^ Two reviewers extracted data from each publication independently. Discrepancies between reviewers’ extracted data were resolved via discussion, or by a third reviewer if necessary. Key extraction variables included publication author and year, study design and methods, setting, target population characteristics, targeted NCD conditions, intervention and delivery characteristics, and quantitative data on intervention coverage and effectiveness. Reported information on delivery barriers and facilitators was also extracted.

### Data synthesis

We summarised and descriptively analysed key characteristics of the populations, interventions and delivery approaches reported in the included publications using tables, frequency plots and maps. No quantitative syntheses (eg, meta-analyses) of intervention coverage or effectiveness were undertaken due to the lack of available data. We narratively synthesised information on delivery barriers and facilitators by classifying author-reported barriers and facilitators with reviewer-generated codes that summarised their content, and then grouping these codes into broader categories or themes.

### Patient and public involvement

Patients and/or the public were not involved in the design, or conduct, or reporting or dissemination plans of this research.

## Results

### Characteristics of included publications

**Figure 1:**
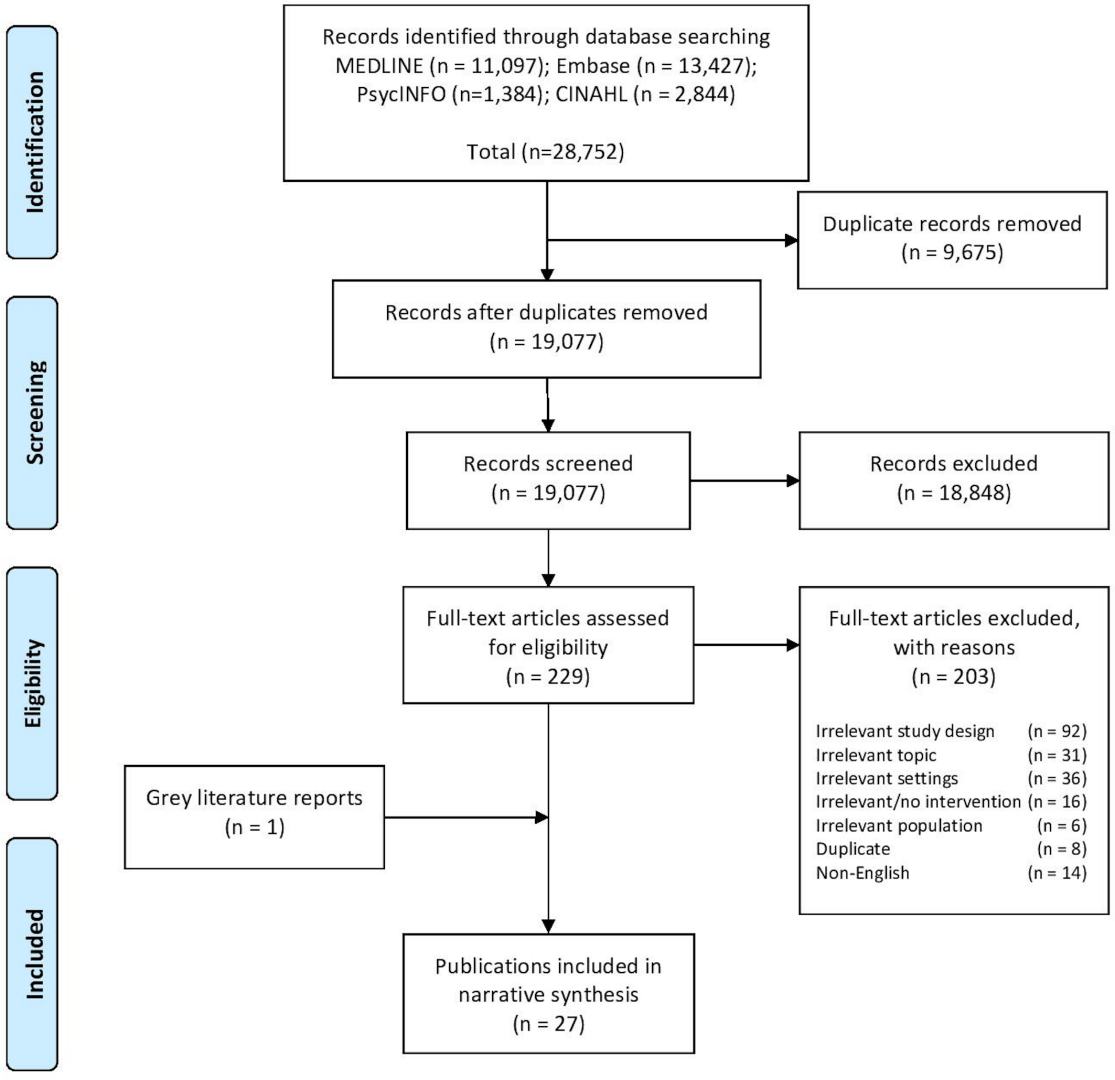
Preferred Reporting Items for Systematic Reviews and Meta-Analysis flow diagram of literature selection.

We identified 19 077 unique records through our indexed database search, 26 of which met the review eligibility criteria (figure 1). One additional eligible publication was identified from our grey literature search, for a total of 27 publications included in this review.^17–43^

**Figure 2:**
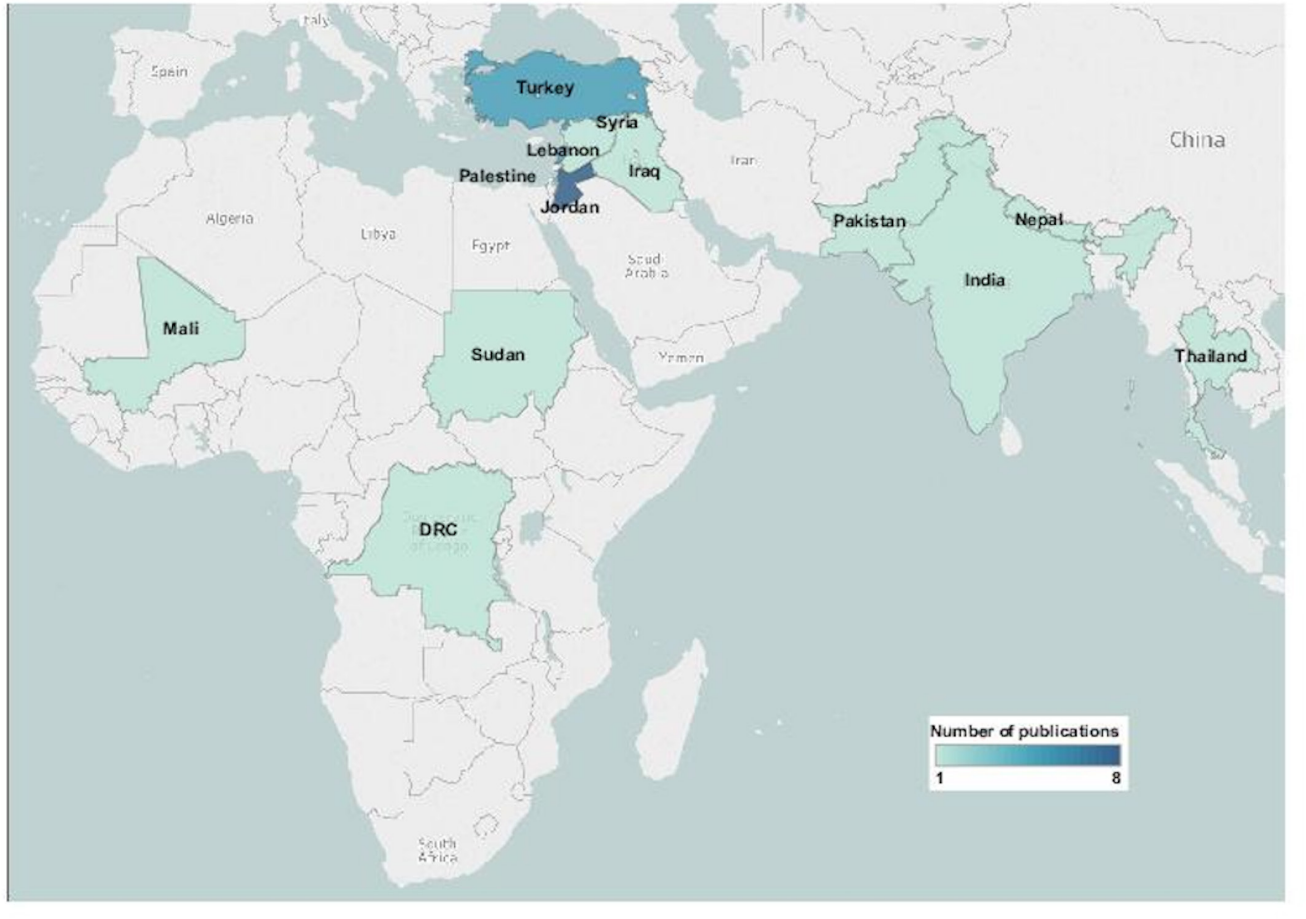
Geographic distribution of included publications.

The key characteristics of each eligible publication are presented in table 1. Almost all publications reported on observational studies, with only one reporting on a randomised controlled trial. Half of the publications reported on studies conducted in countries in the Middle East and North African region (n=14, 52%) (figure 2), with Jordan featuring most frequently (n=8, 30%). Only 13 countries were represented in the included literature overall. The majority of publications reported on interventions delivered to refugees (n=22; 81%). About 30% (n=8) reported on the delivery of interventions targeted at children or adolescents, and only 7% (n=2) on interventions targeted at adult women; the remaining reported on interventions that were delivered to children, adolescents or adult women along with others.

**Table 1:** Characteristics of included publications

Author	Country	Report type	Displacement status	Target population	NCD condition	Intervention	Delivery platform	Delivery site	Delivery personnel
East Asia and Pacific									
Thanapongsathron *et al* 42	Thailand	Observational study	Refugees (camp based)	All	Chronic pain	Surgical treatment	MOH	Hospital	Doctors
Europe and Central Asia									
Akbalik Kara *et al* 19	Turkey	Observational study	Refugees (dispersed)	Children, adolescents	Chronic kidney disease	Dialysis	MOH	Hospital	Doctors
Demir *et al* 27	Turkey	Observational study	Refugees (dispersed)	All	Cardiovascular disease	Surgical treatment	MOH	Hospital	NR
Gucer *et al* 28	Turkey	Observational study	Refugees (dispersed)	Children under 5, adolescents	CFD, haemophilia	Specialised care	NR	Hospital	NR
Hakverdi *et al* 29	Turkey	Observational study	Refugees (dispersed)	All	Leukaemia, other cancers	Surgical treatment	Research	Hospital	NR
Oymak *et al* 36	Turkey	Observational study	Refugees (dispersed)	Children under 5, adolescents	Leukaemia, other cancers	Chemotherapy	MOH	Hospital	NR
Bakkal *et al* 21	Turkey	Observational study	Refugees (dispersed)	All	Breast cancer, other cancers	Chemotherapy, radiotherapy, surgical treatment	MOH	Hospital	NR
Middle East and North Africa									
Abbott *et al* 18	Jordan	Observational study	Refugees (camp based)	Children under 5	Multiple chronic conditions	Screening for referral	NGO/UN agency	Clinic	Doctors
Canali *et al* 24	Jordan	Observational study	Refugees (camp based)	All	Diabetes	Medication	NGO/UN	Clinic	NGO/UN staff
Collins *et al* 25	Jordan	Mixed methods study	Refugees, host (dispersed)	All men and women above 18	COPD and asthma	Medication	NGO/UN agency	Clinic	Doctor, nurses
					Cardiovascular diseases	Medication, diet and lifestyle management, psychosocial support			
Coppola *et a*l^26^	Iraq	Observational study	Civilians (dispersed)	Children, adolescents	Multiple chronic conditions	Surgical treatment	Defence system	Military hospital	Military medical staff
Kallab *et al* 17	Lebanon	Observational study	Refugees, host (dispersed)	All men and women >40	Diabetes, hypertension	Screening, medication	MOH and NGO/UN agency	Mobile clinic, health centre	Doctors
Khader *et al* 32	Jordan	Observational study	Refugees (camp based)	All men and women above 40	Hypertension, diabetes, myocardial infractions, congestive cardiac failure, stroke, renal disorders	Screening for referral	NGO/UN agency	Clinic	Doctors, nurses, support staff
					Hypertension	Medication, diet and lifestyle management			
Khader *et al* 30 31	Jordan	Observational study	Refugees (camp based, dispersed)	All men and women above 40	Diabetes, hypertension, myocardial infractions, congestive cardiac failure, renal disorders	Screening for referral	NGO/UN agency	Clinic	Doctors, nurses, support staff
					Diabetes	Medication, insulin therapy, diet and lifestyle management			
Mousa *et al* 34	Jordan Lebanon Syria Palestine	Observational study	Refugees (dispersed)	All men and women above 40	Diabetes, hypertension	Screening for referral	NGO/UN agency	Clinic	NR
Saab *et al* 38	Lebanon	Observational study	Refugees, host (dispersed)	Children, adolescents	Leukaemia and other cancers	Chemotherapy, radiation, screening and surgical treatment	MOH, NGO/UN agency	Hospital	Doctors
Saadeh *et al* 39	Jordan	Observational study	Refugees (camp based, dispersed)	All men and women above 40	Hypertension	Medication	NGO/UN agency	Clinic	Doctors
Sethi *et al* 40	Lebanon	Observational study	Refugees, host (camp based, dispersed)	All	Multiple chronic conditions	Medications, health education, disease monitoring	NGO/UN agency	Mobile clinic	Doctors, trained refugee outreach workers
Taha *et al* 41	Jordan	Observational study	Refugees (camp based)	Women above 20	Breast cancer	Health education	NGO/UN agency	Home	Trained community outreach workers
				Women above 40		Screening voucher distribution	MOH and NGO/UN agency		
Yusef *et al* 43	Lebanon	Observational study	Refugees (dispersed)	All	Diabetes	Medication, insulin therapy	NGO/UN agency	Clinic	Doctors
					Hypertension	Medication, diet and lifestyle management			
Sub-Saharan Africa									
Ali *et al* 20	Sudan	Observational study	IDPs (dispersed)	Adolescents	Cardiovascular disease	Screening for referral	MOH and NGO/UN agency	Clinic	Doctors
Besancon *et al* 22	Mali	Case study	IDPs, civilians (dispersed)	All	Diabetes	Specialised care with diabetic coma and foot kit	MOH and NGO/UN agency	Hospital	Doctors
Murphy *et al* 35	DRC	Qualitative study	IDPs (dispersed)	All	Diabetes	Medication, diet and lifestyle management, psychosocial support	MOH and NGO/UN agency	Hospital, clinic	Doctor, nurse, nutritionist, support staff
South Asia									
Bhatta *et al* 23	Nepal	Observational study	Refugees and host (camp based, dispersed)	Women above 18	Cervical cancer	Screening for referral	MOH & NGO/UN agency	NR	Doctors
Khan *et al* 33	Pakistan	Observational study	Refugees and host (dispersed)	Children and adolescents under 15	Leukaemia, other cancers	Chemotherapy and radiotherapy	MOH	Hospital	NR
Ryan *et al* 37	India	Randomised control trial	Refugees (dispersed)	All	Arthritis	Chinese medication versus western medication	MOH and research	Clinic	Doctors

All, everyone regardless of age/gender; CFD, congenital factor deficiency; COPD, chronic obstructive pulmonary disease; DRC, Democratic Republic of Congo; IDPs, internally displaced persons; MOH, Ministry of Health; NCD, non-communicable disease; NGO/UN, non-governmental organisations/United Nations; NR, not reported.

### Characteristics of intervention delivery

**Figure 3:**
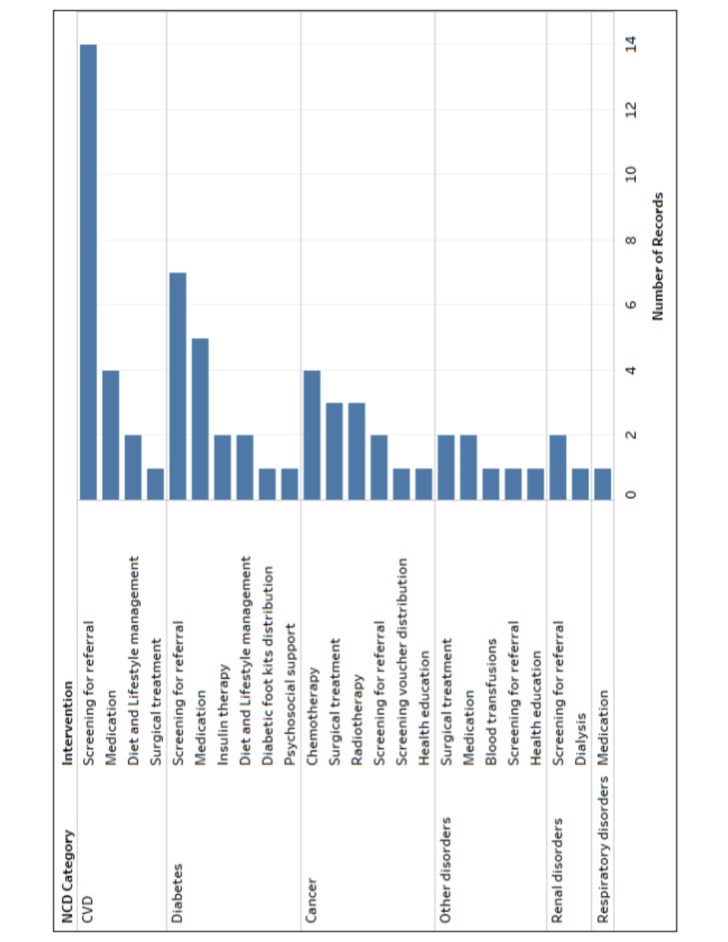
Frequency of reported interventions delivered, by NCD condition. CVD, cardiovascular disease; NCD, non-communicable disease.

The frequencies with which interventions for different NCD conditions were reported to be delivered are presented in figure 3. Cardiovascular disease, diabetes and cancer were the most frequently reported NCDs for which interventions were delivered. Screening was the most frequently reported intervention delivered for cardiovascular disease and diabetes, while chemotherapy was most frequently reported for cancer. Intervention coverage was not reported in any of the publications included in our review, and no publications reported intervention effectiveness estimates for women or children specifically. Below, we synthesise retrieved information on NCD intervention delivery for women and children in conflict settings, organised by level of care: inpatient facility-based delivery, outpatient facility-based delivery, delivery through outreach, and community-based delivery.

#### Inpatient facility-based interventions

Interventions for cancer treatment were most commonly reported as being delivered in inpatient settings, with little information reported on the personnel involved. Four publications reported on the delivery of inpatient cancer treatment to Syrian refugees in government hospitals in Turkey^21 29 36^ and Lebanon^38^ between 2015 and 2018. Thirty-six children with leukaemia and other cancers were treated with chemotherapy at a paediatric hospital in Turkey, with 15 patients admitted for disease relapse.^36^ Most patients were admitted at an advanced stage as they could not access chemotherapy while in Syria due to ongoing war. Other cancer treatments were provided by the Mustafa Kemal University Research Hospital in Hatay district, where 136 tumour cases were reported out of 175 cases who underwent surgery in the Brain Surgery Department.^29^ Another hospital-based retrospective study reported on 134 patients treated for breast and other cancers with chemotherapy, radiation and surgery, between 2015 and 2017 in Sanliurfa Training and Research and Hospital, where 45% of the patients were diagnosed in advanced stage.^21^

In Lebanon, The American University of Beirut Medical Center and the Children’s Cancer Center of Lebanon Foundation, in partnership with St Jude Children’s Research Hospital and the American Lebanese Syrian Associated Charities, established three successive funding programmes to treat displaced children with cancer along with a continuous assessment of resource utilisation. Between 2011 and 2017, 575 Iraqi, Syrian and Palestinian refugee children were evaluated for suspicion of cancer. Of these, 46% (n=311) received direct medical support, 20% (n=107) received full-treatment coverage and 34% children (n=204) had limited-workup, or speciality services.^38^

A further publication, from Pakistan, reported on Afghan refugee and host community children under 15 years of age being provided with hospital-based treatment of leukaemia and other cancers in Peshawar town between 1990 and 1994.^33^

Heart bypass surgery was performed on 53 Syrian refugees between 2012 and 2014 at Sanliurfa Training and Research and Hospital in Turkey, including two emergency surgeries and 51 elective surgeries.^27^ Between 2011 and 2017, two centres caring for Egyptian and Syrian refugee patients in Istanbul provided specialised care to 20 children and adolescents aged 2 to 17 years with congenital factor deficiencies.^28^ Syrian refugee children aged 1 to 17 years old (n=130) with chronic kidney disease were admitted to a tertiary hospital in Gaziantep between September 2012 and January 2015 for paediatric nephrology care. All children were provided with peritoneal dialysis or haemodialysis.^19^

A retrospective cohort study conducted in Iraq reported on 85 injured children treated at a tent-based Level III expeditionary military hospital in Balad during the Iraq war, with military medical staff performing 134 surgeries on 63 children for multiple traumatic and non-traumatic conditions such as congenital, infectious, gastrointestinal and neoplastic causes.^26^ Another study, from Thailand, reported on 122 Cambodian refugees undergoing laparoscopic appendectomy at Borai Hospital in Trat province. Patients presenting with right lower quadrant abdominal pain were referred by a Cambodian doctor from a Thai- Cambodian border refugee camp to the hospital under Royal Thai Army permission.^42^

One publication reported on a non-governmental organisation (NGO)-led initiative for diabetes patients in Mali in March 2012, following a coup in the capital city Bamako. Children with type 1 diabetes were evacuated from the occupied northern regions in Mali to Bamako, and medicines, management tools and other support were provided by doctors to 1814 displaced people with diabetes. Emergency kits prepared by the NGO enabled the care of 32 people with diabetic foot complications and 15 people in diabetic coma.^22^

#### Outpatient interventions

Eleven studies reported interventions conducted in outpatient clinics. Eight of the 11 studies were conducted in the Middle East and North Africa region from the period 1997 to 2016. In 1997, an NCD prevention and control programme run by United Nations Relief and Works Agency for Palestine Refugees in the Near East (UNRWA) in Lebanon and staffed by doctors provided treatment to approximately 4000 diabetic and 3000 hypertensive patients.^43^

A multi-country cross-sectional study conducted among Palestinian refugees in Jordan, Lebanon, Syria, West Bank and Gaza comprised 7762 refugees over 40 years of age who were offered screening for diabetes and hypertension in June 2007.^34^ The screening took place in three UNRWA health centres randomly selected from each area of UNRWA operations, with reported screening coverage ranging from 50% in Syria to 92% in Lebanon. The personnel involved in delivering the intervention were not reported in the study.

One study of Palestine refugees treated by general practitioners at UNRWA primary healthcare clinics (PHCs) in Jordan used existing aggregate procurement data derived from the UNRWA pharmacy records to examine the utilisation of antihypertensive medications between 2008 and 2012.^39^ In 2012, 53 278 patients (14.5% of the served population) aged 40 years and older were diagnosed with hypertension. Despite an increased prevalence of hypertension among patients aged 40 years and older, the number of patients with uncontrolled hypertension significantly declined from 43.3% (n=18 400) to 31.5% (n=16 783) over the study period, a statistically significant decline of about 11.8%.

From 2009, UNRWA also started an electronic health record system (E-Health) at Nuzha PHC and later expanded throughout six of its 24 PHCs in Jordan. Situated in or near Amman, the capital city, the six PHCs served 302 539 refugees in 2012, 26% of the total served population in Jordan. Each clinic is staffed by up to four doctors and a variable number of nurses; all services are provided free of charge. Palestine refugees who attend the clinic are screened annually for diabetes and 6-monthly for hypertension if they are aged 40 years or older, at risk of NCDs or are preconception or pregnant women. Diet and lifestyle management advice is also provided to the patients.^31 32^ The use of the E-Health cohort monitoring system enabled Nuzha PHC Clinic to follow a cohort of over 100 diabetes mellitus patients during a 3-year period and decrease loss to follow-up. The health record system also helps PHC authorities to plan accurately for an ever increasing burden of patients in terms of logistics, laboratory reagents, drugs and staffing levels.

In 2014, MSF started providing free healthcare for NCDs in two outpatient PHCs in northern Jordan, specifically targeting urban Syrian refugees who were dispersed as well as some Jordanians who required access to primary healthcare.^25^ MSF developed their own cardiovascular disease (CVD) risk-based guidelines adapted from WHO PEN.^25^ The PHC clinic accepted patients living with one of five conditions: CVD, hypertension, diabetes, chronic obstructive pulmonary disease or asthma.^25^ Patients with existing CVD (secondary prevention), diabetics aged ≥40, patients with total cholesterol ≥8 mmol/L or patients with WHO/International Society of Hypertension (ISH) risk ≥20%, were eligible for lipid lowering treatment based on WHO PEN.^25^ The healthcare services were offered by nurses and doctors.

In 2016, a cross-sectional survey was conducted by UNRWA to measure the diabetic drug adherence among diabetic patients who attended medical care at the UNRWA Amman Camp health centre in Jordan. Seventy-three per cent of patients (n=557) were non-compliant with the diabetes drug therapy due to use of multiple providers of care and diabetes medications.^24^

In the beginning of 2017, Syrian American Medical Society completed a short-term medical mission to Jordan. These providers visited many sites, including the 5-year-old Zaatari refugee camp positioned just south of the Syrian border. The Zaatari camp was hosting nearly 80 000 displaced Syrians, and 27% were children under 18 years of age.^18^ These refugees received medical care from a variety of NGOs operating within the Zaatari refugee camp, but they sometimes received referrals for healthcare in Jordanian communities outside the camp. During the two days of data collection, the physician diagnosed an average of 69 paediatric patients per day. With a primary diagnosis of upper respiratory infection (n=57), 42 patients (73.6%) received a prescription for paracetamol and 19 patients (33.3%) received a prescription for dextromethorphan; only two of these patients (3.5%) received a prescription for amoxicillin.^18^

In sub-Saharan Africa, studies reported clinic-based interventions in Democratic Republic of Congo (DRC)^35^ and Sudan.^20^ A qualitative study reported a new model of diabetes healthcare (Integrated Diabetic Clinic within an Outpatient Department (IDC-OPD)) implemented by MSF in Mweso Hospital in eastern DRC. Outpatient clinical procedures involved new patient assessment, monthly nurse-led follow-up appointments and 6-month medical review. The service was managed by a nurse supervisor. The staff included a nursing assistant; two doctors who provided medical support and saw referred patients; a nutritionist; an information, education and counselling officer and a psychosocial support officer.^35^ The qualitative findings from focus group discussions of patients and staff emphasised the value of treatment support, including psychosocial and educational support to diabetic patients and their families, and culturally sensitive, low-cost dietary advice, to ensure the adoption and maintenance of diabetes treatment.^35^

In Sudan, a prospective epidemiological study was conducted from July 2016 to September 2016 among internally displaced adolescents living in Nyala camps of South Darfur. Trained medical officers conducted echocardiographic screening for rheumatic heart disease among 1515 cases. Training of health personnel was conducted through lectures.^20^

A randomised controlled trial conducted in an outpatient research clinic among 28 Tibetan refugees in Nepal tested doctor-provided treatment for arthritis with western or traditional Tibetan medication to assess their relative efficacy.^37^ This trial demonstrated a statistically significant improvement (p<0.001) in limb mobility among Tibetan refugees using traditional treatment as compared with western treatment.^37^

#### Outreach Interventions

Only two studies reported mobile clinic-based interventions, conducted in Lebanon among the Syrian refugee and Lebanese host populations in Bekaa Valley. In 2014, Syrian refugees were served in 32 informal settlements through mobile medical clinics providing clinical consultations, medications, disease monitoring, health education and referrals to supported PHC facilities for diagnostic tests and children’s dental care. During the two years of the mobile medical project in Lebanon, local clinicians managed the care of 2000 NCD patients from 120 informal settlements with more than 18 000 consultations; delivered almost 54 000 prescription medications; screened 10 500 children for dental problems and facilitated acute dental care for 1450 children.^40^ This intervention package also included health promotion activities through trained refugee outreach volunteers.^40^ Similarly, another programme was implemented by HelpAge International and Handicap International in 2013 among 3202 Syrian refugees in North Bekaa, West Bekaa, Tyr and Beirut. The programme included screening for diabetes and hypertension according to WHO guidelines at the mobile units free of charge. The clinical assessment was performed by a general practitioner and patients were referred to a specialist when needed, usually a cardiologist. Medications were provided either on a monthly basis or quarterly for stable patients, those with disabilities and those aged 60 plus with reduced mobility, in order to minimise transportation issues.^17^

#### Community-based Interventions

Only one study reported on community-based activities, describing home visits by local community outreach workers providing education about breast cancer and breast health to about 2400 refugee women in Jordan. In addition, mammography screening vouchers were distributed to eligible women aged 40 or older. Out of 625 women that received a voucher for free mammography screening, 73% attended the mammography unit. Women who received a follow-up visit were more likely to use the free mammography voucher compared with those who were not followed-up (83% vs 67%; p<0.001).^41^

#### Barriers to and facilitators of NCD intervention delivery

Table 2 presents a synthesis of reported delivery barriers and facilitators. Destruction of local health facilities and ongoing war conditions posed challenges to active case-finding and to providing a continuum of care to cancer patients.^29 36^ Migration patterns of refugees also affected treatment adherence.^25^ When patients present late for treatment due to the inability to access a health facility, disease prognoses may be worse.^29^ Insufficient health workers to accommodate massive internal displacement and refugee flows, as well as the interruption of donor supplies and funding were the key logistical constraints reported.^22 34 43^

**Table 2:** Synthesis of reported NCD intervention delivery barriers and facilitators

**Barriers**	
*Themes*	*Specific barriers*
Limited population access (n=6)	Population movement^25^ Long travel distance to access facility^35^ Inability to access care and unaffordable services^29^ ^28 29^ ^36^
Logistical constraints (n=3)	Inadequate supplies of commodities^22^ ^34^ ^43^ Inadequate workforce^22 34^ Limited funding^22 34^
**Facilitators**	
*Themes*	*Specific facilitators*
Innovative multidisciplinary model of care (n=1)	The integrated diabetic clinic within an outpatient department^35^
Integration of NGO services into local healthcare system (n=1)	Integration of services through public primary healthcare centres^40^
Capacity building of workforce (n=1)	Training of grassroots leaders (female outreach workers)^41^
Innovative technology use (n=3)	E-Health technology in routine primary healthcare services (The ‘DOTS’ cohort monitoring system)^30–32^

E-Health, electronic health record system; NCD, non-communicable disease; NGO, non-governmental organisations.

Apart from the challenges, some studies reported on various strategies to effectively deliver NCD care, including the implementation of a multidisciplinary model of care. MSF’s IDC-OPD in DRC, for example, is a nurse-led, multidisciplinary model for providing diabetes care in a simplified context of adapted clinical guidelines and standard operating procedures, adapted patient counselling and support materials and staff training by a diabetologist.^35^ In Lebanon, due to the lack of universal healthcare and a system dominated by private health service providers, some vulnerable low-income host Lebanese communities were initially not eligible for the same health subsidies as refugees. To reduce those disparities, recent crisis response plans have emphasised integration of services through public PHCs.^40^ Another innovative strategy reported by a UNRWA working in Jordan involved the adoption of E-Health technology by primary healthcare services to strengthen the monitoring of patients accessing NCDs care.^30–32^ This strategy enabled implementers to better forecast demands for drugs and other consumables and other logistics necessary for providing quality NCDs care as well as tracking the follow-up care of existing patients.^30–32^

## Discussion

### Principal findings

Our review identified 27 publications reporting on the delivery of NCD interventions to conflict-affected women and children in 13 low- and middle-income countries, mostly among refugees displaced in the Middle East and North Africa region and Turkey. Screening and medication for cardiovascular disease and diabetes were the most commonly reported interventions in this literature, with most publications reporting on either inpatient or outpatient facility-based delivery, and very few reporting on delivery through outreach or in the community. Doctors were the most frequently reported delivery personnel.

### Evidence gaps

None of the included publications reported on population-level intervention coverage or on the effectiveness of interventions in improving the health status of conflict-affected women or children. Most of the studies included in this review used a cohort study design, but only a few were able to consistently follow-up participants over time to measure change in NCD biomarkers. Eight studies were cross-sectional in design and four studies were published only as abstracts. Similarly, a previous systematic literature review also found extremely low quality and quantity of evidence about NCD intervention effectiveness in LMICs.^9^

Geographically, the studies predominantly focussed on populations in the Middle East and North Africa region, where high NCD burden is evident, with information on NCD intervention delivery in sub-Saharan Africa and in South Asia available from only three publications in each region, both of which also have high NCD burden.

Children’s access to cancer treatment varied, depending on the country to which refugees were displaced. Syrian refugee children in Turkey had free access to healthcare through the Turkish government,^21 29^ while displaced children with cancer in Lebanon had no means of support from the government and limited support from non-governmental organisations to access treatment.^38^ None of the included studies reported cancer treatment for refugees in Jordan. These findings were similar to those from a recent descriptive review of the ways in which Jordan, Lebanon and Turkey provided NCD services to Syrian refugees over time.^10^ The authors noted the three host countries use different approaches to the design, delivery and financing of NCD services for refugees. In Jordan and Lebanon, a collaborative approach is used to deliver subsidised health services from the Ministries of Health, the UNHCR and other healthcare providers.^10^ In contrast, in sub-Saharan African conflict settings such as in Sudan, Mali and DRC where existing healthcare systems are weak, humanitarian actors provide emergency NCD care to vulnerable populations.^22 35^

Aebischer Perone and colleagues recently presented a series of questions that humanitarian agencies should consider when addressing NCDs in humanitarian crises.^44^ When considering what models of care should be adopted to ensure the continuum and the continuity of care, an integrated, multidisciplinary and interdisciplinary approach is recommended, involving community leaders and other influencers. Our narrative synthesis of barriers and facilitators similarly highlighted the case of the integrated, multidisciplinary model for delivering NCD care in eastern DRC, including activities such as community mobilisation, capacity-building of health staff and the provision of psychosocial support for patients.^35^ The current Sphere Handbook also highlights the need for health worker training for NCDs, as well as the health promotion at the community level;^45^ however, none of the publications included in this review referred to the Sphere Handbook. Aebischer Perone *et al* also flag the need for a robust system for registering and following up enrolled NCD patients, and our findings highlighted the utility of the innovative E-Health system adopted by UNRWA in 2009 and used in PHCs for 4.6 million registered Palestinian refugees in Jordan, Syria, Lebanon, West Bank and Gaza.^34^ This approach includes a web-based, patient-centred application to support UNRWA’s health services for the prevention and management of NCDs such as diabetes, hypertension and other CVDs. The authors reported that the E-Health system improved the overall management of the programme and follow-up care of patients. Other service providers reported a range of challenges to the delivery of NCD care including logistical constraints such as limited medical supplies, insufficient work force and shortage of funding.^22 34 43^ Similar delivery barriers were reported by a previous systematic literature review focussing on Syrian refugee populations.^10^

### Limitations

To the best of our knowledge, this is the first systematic review to examine how NCDs interventions are being delivered to conflict-affected populations, particularly women and children, in low- and middle-income countries. However, in addition to the limited delivery information and effectiveness evidence available from the relevant literature, our review has several other limitations. We included only English publications, and we conducted a comprehensive but not exhaustive search of the grey literature; these methodological decisions may have led to the exclusion of some publications that may have provided different insights into NCD intervention delivery for conflict-affected women and children in LMICs.

### Conclusions and recommendations for future action

Comprehensive management of NCDs is a crucial but neglected aspect of the humanitarian health response in conflict settings. Large and persistent gaps in information and evidence make it difficult to recommend effective strategies for improving the reach of quality NCD care among conflict-affected women and children. Additional research and better reporting of programmatic experience are both urgently needed to help ensure that affordable, high quality NCD interventions are available and accessible at the primary healthcare level for displaced as well as host populations in conflict settings.
