# Supplementary material for: Delivering non-communicable disease interventions to women and children in conflict settings: a systematic review
Source: BMJ Glob Health. 2020 Apr 27;5(Suppl 1):e002047. doi: 10.1136/bmjgh-2019-002047 (PMC7202786; doi:10.1136/bmjgh-2019-002047)
Supplement: Supplementary data [file bmjgh-2019-002047supp001.pdf]

Appendix 1: MEDLINE search strategy

Conflict related terms

1. disasters/ or emergencies/ or mass casualty incidents/
2. disaster victims/
3. ((disaster or disasters or catastrophe or catastrophes) adj5 (environ* or human or manmade or "man made" or nature or natural or weather)).tw,kf.
4. ("mass casualty" or "mass casualties" or "mass fatalities" or "mass fatality").tw,kf.
5. ((crisis or crises) adj5 (environ* or human or manmade or "man made" or nature or natural or weather)).tw,kf.
6. "warfare and armed conflicts"/ or armed conflicts/ or warfare/ or biological warfare/ or bioterrorism/ or chemical warfare/ or chemical terrorism/ or nuclear warfare/ or psychological warfare/ or war crimes/ or ethnic cleansing/ or genocide/ or holocaust/ or war exposure/ or war-related injuries/
7. afghan campaign 2001-/ or gulf war/ or iraq war, 2003-2011/
8. ("afghan campaign" or "armed conflict" or "armed conflicts" or "gulf war" or "iraq war" or "war time" or "wartime").tw,kf.
9. ((armed or zone or political or civil) adj3 (conflict or conflicts or attack or attacks or war or wars or "no fly")).tw,kf.
10. ("war related injuries" or "war related traumas" or "war related injury" or "war related trauma").tw,kf.
11. ("militant group" or "militant groups" or "militant organization" or "militant organizations" or "militant organisation" or "militant organisations").tw,kf.
12. ("biological terrorism" or bioterrorism or biowarfare or "chemical terrorism" or "ethnic cleansing" or "ethnic cleansings" or "gas poisoning" or genocide or holocaust or holocausts or "nuclear terrorism" or "war exposure" or "war exposures").tw,kf.
13. Disaster Medicine/
14. disease outbreaks/
15. Emergency Medical Services/
16. ((emergency or emergencies) adj5 (environ* or human or manmade or "man made" or nature or natural or weather)).tw,kf.
17. Starvation/
18. (famine or famines or starvation or starvations).tw,kf.
19. cyclonic storms/ or droughts/ or floods/ or tornadoes/ or tidal waves/
20. avalanches/ or earthquakes/ or landslides/ or tidal waves/ or tsunamis/ or volcanic eruptions/
21. (avalanche or avalanches or cyclone or cyclones or drought or droughts or earthquake or earthquakes or flood or flooded or flooding or floods or hurricane or hurricanes or landslide or landslides or "land slide" or "land slides" or mudslide or mudslides or "mud slide" or "mud slides" or storm or storms or tornado or tornadoes or tsunami or tsunamis or typhoon or typhoons or "volcanic ash" or "volcanic eruption" or "volcanic eruptions" or "volcanic gases").tw,kf.
22. refugees/

Shah S, *et al. BMJ Global Health* 2020; 5:e002047. doi: 10.1136/bmjgh-2019-002047

23. (evacuee or evacuees or refugee or refugees or squatter or squatters or transients).tw,kf.
24. relief work/ or rescue work/
25. ((rescue or relief or aid) adj (plan or plans or activity or activities or agency or agencies)).tw,kf.
26. ("aid plan" or "aid work" or "relief plan" or "relief work" or "rescue plan" or "rescue work").tw,kf.
27. ((staff or staffs or worker or workers) adj3 (relief or aid)).tw,kf.
28. (humanitarian assistance or humanitarian assistances or relief work or relief works).tw,kf.
29. (humanitarian adj2 (aid or response or relief or crisis or crises or emergency or emergencies or disaster or disasters)).tw,kf.
30. Altruism/
31. (humanitarianism or altruism).tw,kf.
32. ("displaced children" or "displaced families" or "displaced family" or "displaced individuals" or "displaced internally" or "displaced men" or "displaced people" or "displaced peoples" or "displaced person" or "displaced persons" or "displaced population" or "displaced populations" or "displaced women" or "forced displacement" or "forced displacements" or "internal displaced" or "internal displacement" or "internally displaced" or "population displaced" or "population displacement").tw,kf.
33. (((camp or camps) and displac*) or "protected village*").tw,kf.
34. (victim or victims).tw,kf.
35. rubble.tw,kf.
36. or/1-35

Population of interest related terms

37. adolescent/ or young adult/

38. (adolescence or adolescent or adolescents or teen* or youth or youths or "young adult" or "young adults").tw,kf.

39. Pregnant Women/

40. exp pregnancy/

41. (expectant or expectancy or gravid* or pregnant or pregnancies or pregnancy).tw,kf.

42. ("mother to be" or "mothers to be").tw,kf.

43. (prenatal or "pre natal").mp.

44. (perinatal or "peri natal").mp.

45. ((trimester or trimesters) adj3 (first or second or mid or third or final or "1st" or "2nd" or "3rd")).tw,kf.

46. (midtrimester or midtrimesters or "early placental phase" or "early placental phases").tw,kf.

47. exp Delivery, Obstetric/

48. ((labor or labour) adj5 (birth* or breech or childbirth or childbirths or complicat* or difficult or early or easy or induce* or induction or late or obstetric* or onset or pregnan* or present*)).tw,kf.

49. parturients.tw,kf.

50. (birth or births or childbirth or childbirths or parturition or parturitions).tw,kf.

51. ("abdominal deliveries" or "abdominal delivery" or "c-section" or "c-sections" or caesarean or caesareans or cesarean or cesareans or "postcesarean section" or "postcaesarean section").tw,kf.

52. exp Abortion, Induced/

53. (abortion or abortions or embryotomies or embryotomy or "postconception fertility control").tw,kf.

54. ((pregnancy or pregnancies) adj3 terminat*).tw,kf.

55. "sexually active".tw,kf.

56. child/ or child, preschool/ or infant/ or infant, newborn/ or infant, low birth weight/ or infant, small for gestational age/ or infant, very low birth weight/ or infant, extremely low birth weight/ or infant, postmature/ or infant, premature/ or infant, extremely premature/

57. (infan* or newborn* or "new born*" or neonat* or baby* or babies or toddler* or boy or boys or boyfriend or boyhood or girl* or kid or kids or child* or pediatric* or paediatric* or peadiatric* or prematur* or preterm*).mp. or school*.tw.

58. refugees/

59. (refugee or refugees).tw,kf.

60. or/37-59

61. 36 and 60

Domain specific terms – Non-communicable diseases

62. chronic disease/ or multiple chronic conditions/ or noncommunicable diseases/

63. (ncd or "noncommunicable disease*" or "non communicable disease*" or "non infectious disease*" or "noninfectious disease*").tw,kf.

64. ("chronic condition*" or "chronic disease*" or "chronic health condition*" or "chronic medical condition*" or "chronic* ill*" or "long term condition*" or "multi morbidit*" or multimorbidit*).tw,kf.

65. Autoimmune Diseases/

66. ("auto aggressive disease*" or "auto antibody disease*" or "auto immune disease*" or "auto immune disorder*" or "auto immune disturbance*" or "auto immune patholog*" or "auto immuno disease*" or "auto immunologic disease*" or "autoaggressive disease*" or "autoantibody disease*" or

Shah S, *et al. BMJ Global Health* 2020; 5:e002047. doi: 10.1136/bmjgh-2019-002047

"autoimmune disease*" or "autoimmune disorder*" or "autoimmune disturbance*" or "autoimmune patholog*" or "autoimmuno disease*" or "autoimmunologic disease*").tw,kf.

67. lupus erythematosus, systemic/ or lupus nephritis/ or lupus vasculitis, central nervous system/

68. ("brain angiitis" or "brain arteritis" or "central nervous system lupus" or "cerebral arteritis" or "cerebral vasculitis" or "disseminated lupus" or "erythematodes visceralis" or "libman sacks disease" or "lupoid nephritis" or "lupovisceritis" or "lupus erythematodes disseminatus" or "lupus erythematosus disseminatus" or "lupus erythematosus visceralis" or "lupus glomerulonephritides" or "lupus glomerulonephritis" or "lupus glomerulonephritis" or "lupus kidney" or "lupus meningoencephalitides" or "lupus meningoencephalitis" or "lupus nephritides" or "lupus nephritis" or "lupus nephropathy" or "osler libman sacks disease" or "s.l.e." or "sle" or "systemic lupus erythematodes" or "systemic lupus erythematosis" or "systemic lupus erythematosu" or "systemic lupus erythematous").tw,kf.

69. Cardiovascular Diseases/

70. ("angiocardiovascular disease*" or "cardiovascular complication*" or "cardiovascular disease*" or "cardiovascular disorder*" or "cardiovascular disturbance*" or "cardiovascular lesion*" or "cardiovascular syndrome" or "cardiovascular vegetative disorder*" or "major adverse cardiovascular event*").tw,kf.

71. Heart Diseases/

72. ("cardiac anomal*" or "cardiac disease*" or "cardiac disturbance*" or "cardiopath*" or "heart deficienc*" or "heart deformit*" or "heart disease*" or "heart disorder*" or "heart dysfunction*").tw,kf.

73. Myocarditis/

74. ("cardiac inflammation" or carditis or "heart inflammation" or myocarditides or myocarditis).tw,kf.

75. Cardiomyopathies/

76. (cardiomyopath* or "heart myopath*" or "myocardial disease*" or myocardiopath*).tw,kf.

77. Arrhythmias, Cardiac/

78. (arrhythmia* or "cardiac dysrhythmia*" or "cardial arrhythmia*" or "ectopic heart rhythm*" or "ectopic rhythm*" or "heart aberrant conduction*" or "heart dysrhythmia*" or "heart ectopic beat" or "heart ectopic ventricle contraction*" or "heart rhythm disorder*").tw,kf.

79. exp Angina Pectoris/

80. (angina or "anginal attack" or anginas or "angor pectoris" or "coronary spasm*" or "coronary vasospasm*" or "myocardial preinfarction syndrome" or "myocardial preinfarction syndromes" or stenocardia or stenocardias or "syndrome x" or "x syndrome").tw,kf.

81. Myocardial Infarction/

82. ("cardiac infarc*" or "cardial infarc*" or "cardiovascular stroke*" or "heart attack*" or "heart infarc*" or "heart micro infarc*" or "heart muscle infarc*" or "myocardial infarc*" or "myocardium infarc*" or "premonitory infarc* sign" or "second heart attack*" or "subendocardial infarc*").tw,kf.

83. Coronary Disease/

84. (chd or "coronary disease*" or "coronary heart disease*").tw,kf.

85. Heart Failure/

86. ("cardiac backward failure*" or "cardiac decompensation*" or "cardiac failure*" or "cardiac incompetence*" or "cardiac insufficienc*" or "cardiac stand still" or "cardial decompensation*" or "cardial insufficienc*" or "decompensatio cordis" or "heart backward failure*" or "heart decompensation*" or "heart failure*" or "heart incompetence*" or "heart insufficienc*" or "insufficientia cardis" or "myocardial failure*" or "myocardial insufficienc*").tw,kf.

87. Hypertension/ or Renal Hypertension/ or Hypertension, Renovascular/

88. ("Goldblatt hypertension" or "Goldblatt kidney" or "high blood pressure*" or "hypertension" or "hypertensive disease*" or "hypertensive effect*" or "hypertensive response*").tw,kf.

89. Ischemia/

90. ("blood circulation disorder*" or "blood flow disorder*" or "circulation disorder*" or "circulation failure*" or "circulatory disorder*" or "circulatory disturbance*" or "circulatory failure*" or ischaemia* or "ischaemic disease*" or "ischaemic syndrome" or ischemia* or "ischemic disease*" or "ischemic syndrome").tw,kf.

91. Cerebrovascular Disorders/

92. ("brain angiopath*" or "brain circulation failure*" or "brain vascular disease*" or "brain vascular disorder*" or "brain vasculopath*" or "cerebral small vessel disease*" or "cerebral vascular disease*" or "cerebral vascular disorder*" or "cerebral vascular disturbance*" or "cerebral vascular lesion*" or "cerebral vasculopath*" or "cerebrovascular damage*" or "cerebrovascular disease*" or "cerebrovascular disorder*" or "cerebrovascular insufficienc*" or "cerebrovascular lesion*" or "cerebrovascular occlusion*" or "cerebrovascular pathology*" or "cerebrovascular syndrome*" or "intracranial vascular disease*" or "intracranial vascular disorder*").tw,kf.

93. Stroke/

94. (apoplexia or apoplexy or "basal ganglia cerebrovascular disease*" or "basal ganglion hemorrhage*" or "bow hunter syndrome" or "brain accident*" or "brain attack*" or "brain blood flow disturbance*" or "brain hemangioma*" or "brain hematoma*" or "brain hemorrhage*" or "brain infarc*" or "brain insult*" or "brain ischaemic attack*" or "brain ischemia*" or "brain ischemic attack*" or "brain vascular accident*" or "carotid artery disease*" or "central nervous system vasculitis" or "cerebral artery disease*" or "cerebral insult*" or "cerebral small vessel diseases" or "cerebral stroke*" or "cerebral vascular accident*" or "cerebral vascular insufficienc*" or "cerebro vascular accident*" or "cerebrovascular accident*" or "cerebrovascular arrest*" or "cerebrovascular failure*" or

"cerebrovascular injur*" or "cerebrovascular insufficienc*" or "cerebrovascular insult*" or "cerebrovascular malformation*" or "cerebrovascular trauma*" or "cerebrum vascular accident*" or "dementia, vascular*" or "intracranial aneurys*" or "intracranial arterial diseases" or "intracranial arteriovenous malformations" or "intracranial embolism and thrombosis" or "intracranial hemorrhages" or "intracranial vasospasm" or "ischaemic cerebral attack*" or "ischaemic seizure*" or "ischemic cerebral attack*" or "ischemic seizure*" or "melas syndrome" or "occlusive cerebrovascular disease*" or "ocular ischemic syndrome*" or "posterior reversible encephalopathy syndrome*" or "sneddon syndrome*" or stroke* or "susac syndrome" or "vascular headaches" or "vertebrobasilar insufficienc*" or "wallenberg syndrome").tw,kf.

95. Hypercholesterolemia/

96. (cholesteremia or cholesterinemia or "elevated cholesterol" or "elevated cholesterols" or "high cholesterol level" or "high cholesterol levels" or hypercholesteremia or hypercholesteremias or hypercholesterinaemia or hypercholesterinemia or hypercholesterolaemia or hypercholesterolemia or hypercholesterolemias).tw,kf.

97. Cholesterol/

98. (cholesterin or cholesterine or cholesterol or epicholesterol).tw,kf.

99. Blood Pressure/

100. ("blood pressure*" or "blood tension" or "diastolic pressure*" or "intravascular pressure" or normotension or "pulse pressure*" or "systolic pressure*" or "vascular pressure*").tw,kf.

101. Blood Glucose/

102. ("blood glucose" or "blood serum glucose" or "blood sugar" or glucosaemia or glucosemia or "glycaemia" or glycemia or normoglycaemia or normoglycemia or "plasma glucose" or "serum glucose" or "serum sugar").tw,kf.

103. diabetes mellitus/ or diabetes mellitus, type 1/ or diabetes mellitus, type 2/ or diabetes mellitus, lipoatrophic/

104. (diabetes or diabetic* or "dm 1" or "dm 2" or iddm or "mckusick 22210" or mody or niddm).tw,kf.

105. Obesity/

106. ("adipose tissue hyperplasia" or adipositas or adiposity or "fat overload syndrome" or obese or obesitas or obesity or "over weight" or overweight).tw,kf.

107. Anemia/

108. (anemia or anemias or anaemia or anaemias).tw,kf.

109. Neoplasms/

110. (cancer or cancers or malignanc* or neoplas* or tumor or tumors or tumour or tumours).tw,kf.

111. Asthma/

112. (asthma* or "lung allergy").tw,kf.

113. Pulmonary Disease, Chronic Obstructive/

114. ("chronic airflow obstruction*" or "chronic airway obstruction*" or "chronic obstructive airway disease*" or "chronic obstructive bronchitis" or "chronic obstructive bronchopulmonary disease*" or coad or copd or copd or "lung chronic obstructive disease*" or "obstructive lung disease*" or "obstructive lung disorder*" or "obstructive pulmonary disease" or "obstructive pulmonary disorder*" or "obstructive respiratory disease*" or "obstructive respiratory tract disease").tw,kf.

115. Bronchitis/

116. ("bronchial infection*" or "bronchitic syndrome" or bronchitides or bronchitis or "bronchus infection*" or "bronchus inflammation*" or "bronchus mucosal inflammation*").tw,kf.

117. ("pulmonary function" or "lung function").tw,kf.

118. Lung Diseases/

119. ("bronchopulmonary disease*" or "lung chronic disease*" or "lung disease*" or "lung disorder*" or "lung suppurative disease*" or "pleuropulmonary disease*" or "pneumonosis" or "pneumopathy" or "pulmonary disease*" or "pulmonary disorder*" or "silo filler disease" or "silo filler's disease").tw,kf.

120. Renal Insufficiency/

121. ("kidney failure*" or "kidney insufficienc*" or "renal failure*" or "renal insufficienc*").tw,kf.

122. Renal Insufficiency, Chronic/

123. ("chronic kidney disease" or "chronic kidney diseases" or "chronic renal disease" or "chronic renal diseases" or "chronic nephropathy*" or "kidney chronic failure" or "ckd").tw,kf.

124. (hepatic function or hepatocyte function or "liver function").tw,kf.

125. Liver Diseases/

126. ("hepatic disease*" or "hepatic disorder*" or hepatopath* or "liver cell disease*" or "liver disease*" or "liver disorder*" or "liver dysfunction*" or "liver illness*").tw,kf.

127. Liver Cirrhosis/

128. (cirrhosis or "liver fibroses" or "liver fibrosis").tw,kf.

129. Osteoporosis/

130. ("age related bone loss*" or osteoporoses or osteoporosis or "osteoporotic decalcification").tw,kf.

131. Fibromyalgia/

132. ("diffuse myofascial pain syndrome" or fibromyalgia or fibromyalgias or "fibrositic nodule" or fibrositides or fibrositis or fibrositis or "muscular rheumatism").tw,kf.

133. Musculoskeletal Diseases/

134. ("musculo skeletal complaint*" or "musculo skeletal disease" or "musculo skeletal diseases" or "musculo skeletal disorder*" or "musculo skeletal symptom*" or "musculoskeletal complaint*" or "musculoskeletal disease" or "musculoskeletal diseases" or "musculoskeletal disorder*" or "musculoskeletal symptom*" or "orthopaedic disorder*" or "orthopedic disorder*").tw,kf.

135. Chronic Pain/

136. ("chronic intractable pain" or "chronic intractable pains" or "chronic pain" or "chronic pains").tw,kf.

137. Arthritis/

138. (arthritides or arthritis or polyarthritides or polyarthritis or arthrochondritis or arthrosynovitis or "joint inflammation" or oligoarthritis).tw,kf.

139. Cystic Fibrosis/

140. ("cystic fibrosis" or "fibrocystic disease" or "fibrocystic diseases" or "mckusick 21970" or mucoviscidosis or mucoviscoidosis or "pancreas cystic disease" or "pancreas fibrosis" or "pancreatic fibrosis").tw,kf.

141. Endocrine System Diseases/

142. ("endocrinal disorder*" or "endocrine disease*" or "endocrine disorder*" or "endocrine disturbance*" or "endocrine dysfunction*" or "endocrine gland disease*" or "endocrine syndrome*" or "endocrine system disease*" or "endocrinological disease*" or "endocrinopath*" or "hormonal disorder*" or "hormone dysfunction*" or "hormone imbalance*").tw,kf.

143. Thyroid Diseases/

144. ("thyroid abnormalit*" or "thyroid anomal*" or "thyroid disease*" or "thyroid disorder*" or "thyroid dysfunction*" or "thyroid gland disease*" or "thyroid gland dysfunction*").tw,kf.

145. Nervous System Diseases/

146. ("autoimmune diseases of the nervous system" or "nervous disease*" or "nervous disorder*" or "nervous system disease*" or "nervous system disorder*" or "neural disease*" or "neurogenic disease*" or "neurologic complaint*" or "neurologic disorder*" or "neurologic disturbance*" or "neurologic dysfunction*" or "neurologic manifestations" or "neurologic sign*" or "neurologic symptom*" or "neurologic syndrome*" or "neurological complaint*" or "neurological deficienc*" or "neurological disease*" or "neurological disorder*" or "neurological disturbance*" or "neurological sign*" or "neurological symptom*" or "neurological syndrome*").tw,kf.

147. Parkinson Disease/

148. ("paralysis agitans" or "paralysis agitans" or "parkinson dementia complex" or "parkinson disease" or "parkinsons disease" or "parkinson's disease" or "primary parkinsonism").tw,kf.

149. Colitis/

150. (colitides or colitis or "colon inflammation" or "colon inflammatory disease*").tw,kf.

151. Multiple Sclerosis/

152. ("chariot disease" or "disseminated sclerosis" or "insular sclerosis" or "multiple sclerosis" or "sclerosis multiplex").tw,kf.

153. Alzheimer Disease/

154. (alzeimers or alzheimer or "alzheimer's" or "diffuse cortical sclerosis" or "presenile dementia" or "primary senile degenerative dementia" or "senile dementia").tw,kf.

155. or/62-154

156. 61 and 155

157. limit 156 to ed=20180401-20181231

158. ("2018 04*" or "2018 05*").dt.

159. 156 not (157 or 158) [exclude records added after Mar 31, 2018]

~~~ End of Appendix ~~~

Shah S, *et al. BMJ Global Health* 2020; 5:e002047. doi: 10.1136/bmjgh-2019-002047
~~~
